# Final height in Italian patients with congenital hypothyroidism detected by neonatal screening: a 20-year observational study

**DOI:** 10.1186/s13052-015-0190-y

**Published:** 2015-10-28

**Authors:** Maurizio Delvecchio, Maria Cristina Vigone, Malgorzata Wasniewska, Giovanna Weber, Rosa Lapolla, Pietro Pio Popolo, Giulia Maria Tronconi, Raffaella Di Mase, Filippo De Luca, Luciano Cavallo, Mariacarolina Salerno, Maria Felicia Faienza

**Affiliations:** Section of Pediatrics, Department of Biomedical Sciences and Human Oncology, University of Bari Aldo Moro, Bari, Italy; Department of Pediatrics Endocrinology, IRCCS Vita-Salute San Raffaele University, Milan, Italy; Department of Pediatrics, University of Messina, Messina, Italy; Department of Pediatrics, University of Foggia, Foggia, Italy; Pediatric Section-Department of Translational Medical Sciences, University of Naples Federico II, Naples, Italy

**Keywords:** Congenital Hypothyroidism, Secular trend, Thyroid, Growth, Final height, Obesity

## Abstract

**Background:**

Linear growth and final height are reported as normal in congenital hypothyroid patients in the neonatal screening era.

**Methods:**

We evaluated the final height in 215 patients with congenital hypothyroidism to assess if it improved over the last 2 decades.

**Results:**

Final height (-0.1 ± 1.0 SDS) was higher than target height (-0.8 ± 1.0 SDS, *p* < 0.001) and not different among the 4 quartiles for birthdate. It was correlated with target height (r^2^ = 0.564, *p* < 0.001) and height at puberty onset (r^2^ = 0.685, *p* < 0.001), but not with age at diagnosis or the starting LT4/kg/day dose. The curve fitting analysis showed that the age at diagnosis progressively decreased during the 20-year study period, while the target height and the starting LT4/kg/day increased. Final height was not affected by the birthdate, the age at diagnosis, the starting LT4 dose.

**Conclusions:**

The final height is higher than the target height, but despite the improvement in the screening and the treatment, it did not improve over the last 20 years. These findings are in keeping with the described secular trend and suggest that earlier diagnosis and replacement therapy do not significantly modify final height in these patients.

## Introduction

Congenital hypothyroidism (CH) is the most frequent endocrine disease in childhood which causes mental delay and short stature if untreated [1, 2]. The neonatal screening allows early diagnosis while appropriate treatment with replacement dose of levo-thyroxine (LT4) prevents its consequences. Since 70s, when neonatal screening for CH was established in most of the developed Countries, the mean age at diagnosis has been progressively reduced and the auxological and neurological outcomes significantly improved.

Linear growth, onset and progression of puberty, pubertal growth and final height (FH) are reported as rather normal in patients diagnosed thorough the screening, and the target height (TH) is the most important factor determining linear growth [3–8]. The neurodevelopmental outcome is greatly superior in infants detected through neonatal screening than in those diagnosed after the onset of clinical signs, and CH severity at diagnosis, the initial LT4 dose and the timing of normalization of thyroid function are the main factors to prevent mental delay [9–16]. However, even if the neonatal screening programs ensure the prompt initiation of the therapy and the neurodevelopmental outcome can be considered generally normal, some problems in relation to mental outcome are still reported in follow-up studies [17, 18]. Recent papers focusing on educational attainment, cognitive and motor outcome failed to show that in young CH adults diagnosed in the screening era the age at starting treatment plays a key-role in educational attainment [19, 20].

The effects of the improvement in early diagnosis and treatment on neurodevolopmental outcome have been clearly documented by several studies [21], while this relationship has not yet been proved as concern growth and pubertal outcome.

A significant relationship between the age at the start of treatment and FH was demonstrated [5], but it was not confirmed by the correlation analysis performed in a larger study population [7].

The studies focusing on linear growth were run in patients diagnosed in the early CH screening era, i.e. late 70s and 80s, when the age at starting treatment was higher and the starting LT4 dose lower than in 90s and later on. Prompted by these data, we evaluated the FH in a large cohort of patients with permanent CH aiming to evaluate whether the earlier diagnosis and the higher L-T4 starting dose lead to an improvement in growth and pubertal outcome over the last 2 decades.

## Methods

### Patients

In this multicentre observational study, we enrolled 215 patients (152 females, 63 males) with permanent CH, born at full term after an uncomplicated pregnancy between 1980 and 2000 and referred to our Hospitals because of positive neonatal screening. The diagnosis was confirmed by serum TSH, T4 and/or free T4 levels at a mean age of 25.1 ± 10.5 days. The aetiology was assessed in all the patients by a 99mTc-pertechnetate thyroid scan performed in the same morning of the blood sampling for confirmatory diagnosis. An additional thyroid ultrasound was performed in all the patients with athyreosis. Forty-four patients (20.5 %) showed an *in situ* thyroid, 109 (50.7 %) an ectopic gland, 62 (28.8 %) were athyreotic. On the day of diagnosis, oral treatment with LT4 was started at a mean dose of 8.8 ± 2.9 mcg/kg/day. Patients with transient CH, or not identified by the screening program, and CH patients with conditions that could affect growth (celiac disease, small for gestational age, chromosopathy, etc.) or with low compliance to the replacement treatment during the follow up were excluded from the study. The study was approved by the local Ethic Committee.

### Follow-up

The patients assumed regularly the daily LT4 dose 20-30 min before the first meal of the day. The dose was titrated on the basis of clinical and biochemical findings in order to maintain a satisfactory health condition and to keep the TSH and the free T4 within the normal limits. A complete physical and auxological evaluation, and hormonal levels assays were performed regularly in keeping with guidelines. FH was defined as growth velocity < 1 cm/year. The follow-up period at FH attainment was 16.1 ± 1.7 years. 

We recorded age and LT4 dose at diagnosis, age and height at puberty onset, TH and FH. Patients’ and their parents’ standing height was measured by a wall-mounted Harpenden Stadiometer. TH was calculated as follow: (mother’s height + father’s height) + 6.5 cm (if males) or - 6.5 cm if females [22]. The onset of puberty was defined in males by testicles volume 4 ml and in females by breast development 2nd Tanner stage. The onset of puberty was defined as normal if between 8.0 and 13.4 yrs in females and 9.0–14.0 yrs in males [23]. Hormonal assays were performed using commercial kits by radioimmunoassay until 1995 and by immunofluorescence assay thereafter.

### Statistical analysis

Height was calculated as standard deviation score (SDS) in keeping with the Italian standard growth charts [24]. The rate of overweight and obesity was calculated on the basis of the Italian growth charts [24]. The difference between FH and TH was calculated both in cm and in SDS and defined as ∆height. To further evaluate if FH increased over the years, the patients were classified into 4 subgroups on the basis of the quartile for birthdate. Data were expressed as mean ± standard deviation (SD) and the statistical analysis was performed with SPSS computer software for Windows (version 17.5, SPSS Inc.). The t-student test for paired data was used to compare FH, TH, and H at puberty onset. The Analysis of Variance (ANOVA) and the Chi-Square test were used to compare mean values and frequencies among groups, respectively. The correlation coefficients were determined by the Pearson’s test. The prediction model of FH was developed by means of multiple linear regression analysis with a stepwise method fitted by least squares with the following variables: 1) TH SDS; 2) H at onset of puberty; 3) age at diagnosis; 4) starting LT4/kg/day. The curve fitting regression analysis with linear model was used to evaluate the relationship between parameters. All the differences with p - value < 0.05 were considered statistically significant.

## Results

### Puberty onset

**Fig. 1:**
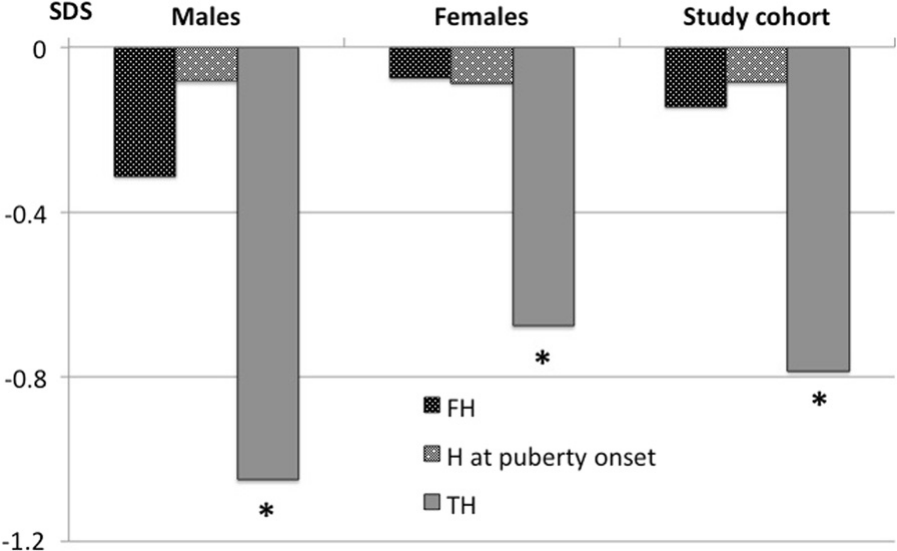
HEIGHT. The figure displays the main values of final height (FH), height (H) at puberty onset, and target height (TH) in males, females, and in whole study cohort. * vs FH: *p* < 0.001. * vs H at puberty onset: *p* < 0.001

The puberty onset occurred in males at 11.4 ± 1.2 years, in females at 10.4 ± 1.1 years. It was within the normal limits for age in 212 (98.6 %) patients, one male showed anticipated puberty (8.5 years), and 1 male and 1 female delayed puberty (14.5 and 13.9 years, respectively). The age at puberty onset was not different among the four quartiles for birthdate in males and in females. Height at puberty onset was -0.1 ± 1.0 SDS and the height gain during puberty was -0.1 ± 0.8 SDS, both of them not different between the 3 etiological groups or between males and females (Table 1). Height at puberty onset was significantly higher than TH (*p* < 0.001) (Fig. 1).

**Table 1:** The table displays the population size and mean ± standard deviation of age at diagnosis, LT4 starting dose, height (H) at puberty onset, heigth gain during puberty, final height (FH), and target height (TH) in the recruited patients classified as etiology and gender. ^a^ vs males: *p* = 0.009

	*In situ* thyroid	Ectopic gland	Athyreosis	Males	Females	Study cohort
Number of patients (%)	44 (20.5 %)	109 (50.7 %)	62 (28.8 %)	63 (29.3 %)	152 (70.7 %)	215 (100 %)
Age at diagnosis (days)	25.4 ± 10.1	24.1 ± 6.8	26.6 ± 15.1	24.7 ± 11.7	25.2 ± 10.0	25.1 ± 10.5
LT4 starting dose (μg/kg/day)	8.9 ± 2.8	8.8 ± 3.0	8.6 ± 3.0	8.1 ± 2.7	9.1 ± 3.0	8.8 ± 2.9
H at puberty onset (SDS)	-0.3 ± 1.0	0.1 ± 0.9	-0.2 ± 0.9	-0.1 ± 0.9	-0.1 ± 1.0	-0.1 ± 1.0
Pubertal H gain (SDS)	0.1 ± 0.7	-0.2 ± 0.8	0.1 ± 0.8	-0.2 ± 0.8	0.0 ± 0.8	-0.1 ± 0.8
FH (SDS)	-0.2 ± 1.0	-0.1 ± 1.0	-0.1 ± 1.1	-0.3 ± 1.0	-0.1 ± 1.0	-0.1 ± 1.0
TH (SDS)	-1.0 ± 1.0	-0.8 ± 0.9	-0.7 ± 1.0	-1.0 ± 0.8	-0.7 ± 1.0^a^	-0.8 ± 1.0

Data cohort according to aetiology and gender

### Height

#### Final height

FH (-0.1 ± 1.0 SDS) was significantly higher than TH (-0.8 ± 1.0 SDS, *p* < 0.001) (Table 1, Fig. 1) and it was not different as compared to height at puberty onset. FH was 172.5 ± 6.2 cm (-0.3 ± 1.0 SDS) in males and 160.0 ± 6.2 cm (-0.1 ± 1.0 SDS) in females, both of them significantly higher than TH (*p* < 0.001 in both gender). The ∆height was 2.5 ± 5.6 cm in males and 2.3 ± 5.7 cm in females. FH, TH, and ∆height were not different among patients with *in situ* thyroid, or athyreosis, or ectopic gland (Table 1). FH and ∆height were was not different between males and females, while TH was lower in males than in females (-1.0 ± 0.8 vs -0.7 ± 1.0, *p* = 0.009) (Table 1).

#### Quartiles analysis

In the forth quartile, the age at diagnosis was lower than in the first (*p* < 0.001) and in the second one (*p* < 0.005), and the starting LT4 was higher than in the other 3 quartiles (*p* < 0.001) (Table 2). FH was not different among the 4 quartiles, while the TH was significantly higher in the forth quartile than in the first (*p* = 0.006).

**Table 2:** The Table displays mean ± standard deviation of age at diagnosis, starting dose of levo-thyroxine (LT4), height (H) at puberty onset, final height (FH), and target height (TH) in the recruited patients divided in quartiles on the basis of birthdate. ^a^ vs 4 th quartile *p* ≤ 0.001; ^b^ vs 4 th quartile *p* ≤ 0.005; ^c^ vs 1st quartile and vs 2nd quartile *p* < 0.05

	1st quartile, 54 patients (18-Mar-80/9-Nov-84)	2nd quartile, 54 patients (30-Nov-84/23-Jun-90)	3rd quartile, 54 patients (29-Jul-90/27-Jan-95)	4th quartile, 53 patients (27-Feb-95/9-Dec-00)
Age at diagnosis (days)	28.5 ± 11.9 ^a^	27.6 ± 12.6 ^b^	23.4 ± 7.3	20.8 ± 7.4
Starting LT4/kg/day (μg)	7.5 ± 2.4 ^a^	7.6 ± 2.3 ^a^	8.9 ± 2.4 ^a, c^	11.2 ± 3.0
H at puberty onset (SDS)	-0.2 ± 1.0	0.0 ± 1.0	0.0 ± 1.0	-0.1 ± 0.8
FH (SDS)	-0.1 ± 1.1	0.0 ± 1.1	-0.1 ± 1.0	-0.3 ± 0.9
TH (SDS)	-1.0 ± 0.9 ^a^	-0.9 ± 1.2	-0.7 ± 0.8	-0.5 ± 0.9

Data according to quartile for birthdate

#### Correlation and regression analysis

FH was significantly correlated with TH (r^2^ = 0.564, *p* < 0.001) and height at puberty onset (r^2^ = 0.685, *p* < 0.001), but not with age at diagnosis or the starting LT4/kg/day dose. The best equation predicting FH was FH SDS = 0.175 + 0.55 H at onset of puberty SDS + 0.328 TH SDS (*p* < 0.001). This model explains 75.2 % of FH variability, with a standard error of 0.7 SDS. In our model, H at onset of puberty is the most important predictor, accounting for 68.5 % of FH variability itself. TH accounts for 56.4 % of FH variability. Both the age at diagnosis and the starting LT4/kg/day were excluded by the statistical analysis in the prediction model.

**Fig. 2:**
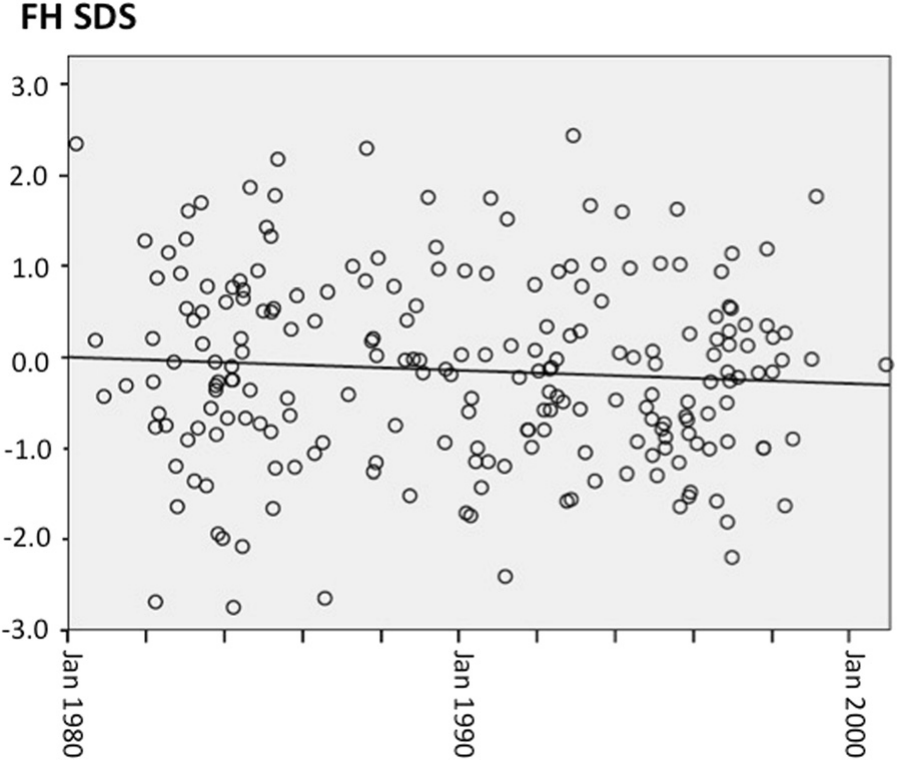
FH AND BIRTHDATE. The figure displays the linear relationship between final height (FH) and birthdate (*p* = ns)

**Fig. 3:**
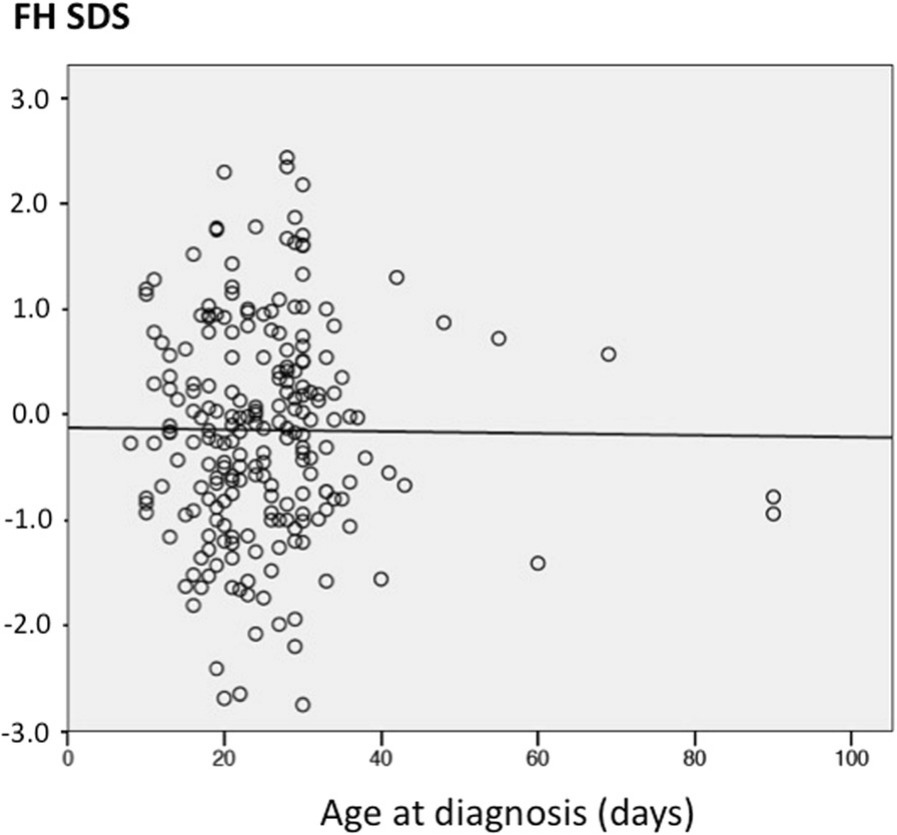
FH AND AGE AT DIAGNOSIS. The figure displays the linear relationship between final height (FH) and age at diagnosis (*p* = ns)

The curve fitting analysis showed that the age at diagnosis progressively decreased (r^2^ = 0.083, *p* < 0.001), while the TH and the starting LT4/kg/day progressively increased (r^2^ = 0.200, *p* < 0.001, and r^2^ = 0.033, *p* = 0.007, respectively) over the study period. FH was not affected by the birthdate (Fig. 2), the age at diagnosis (Fig. 3), nor the starting LT4 replacement dose.

### Overweight and obesity

Sixty-three patients (29.3 %) were overweight and 16 (7.4 %) obese, without any statistical difference on the basis of gender, aetiology, nor quartile for birthdate.

## Discussion

Since it was established in 70s, the CH screening was significantly improved year by year and the current guidelines [16] suggest a starting dose of 10-15 μg/kg/day of LT4, higher than in the past, to prevent the sequelae due to thyroid hormones deficiency. Nowadays, CH patients are more properly treated and it is well acknowledged that the replacement LT4 dose beyond the neonatal period is different on the basis of CH aetiology [25–27].

Neonatal screening programs for CH have been highly successful over the last years in improving both cognitive and growth outcome.

We had already shown that FH in CH patients is normal and significantly higher than TH, and that these 2 parameters are very well correlated among them, without any effect exerted by the age at the start of treatment [7]. However in a previous paper, Bain et al [5] showed that the patients with a positive ∆height had a significantly younger mean age at the start of treatment than children with a negative ∆height. However, the present study confirms our previous data, that the age at the start of treatment does not exert any effect on growth outcome.

To assess if the final height improved over the last 20-years, we divided patients on the basis of birthdate, in quartiles, and we also evaluated all data through the fitting curve analysis. Our data confirmed that FH in CH patients is normal and significantly higher than TH, and that these 2 parameters were very well correlated among them. The age at the start of treatment did not play any role on FH, which was not different among the 4 quartiles of birthdate. The regression analysis confirmed that FH did not change over the 20-year study period (Fig. 2), while TH slowly increased, thus suggesting that the secular trend in height accounts to a large extent for our findings. Since 1850 there has been a positive secular trend in height among European populations [28–30]. In many European countries and in Italy as well, this secular trend slowed down or even reached a plateau since the 80s/90s [30–34] whereas in other Countries, like Belgium, Spain, and Portugal, average heights might still increase [32]. This trend well fits with our data: TH was assessed on parents born in 50s, 60s, and 70s, when the height steadily raised up, while the siblings were born when the secular trend slowed down or reached a plateau. Reasonably, FH does not improve over the 20-year study in keeping with the secular trend. The lack of relationship between the birthdate and FH and between the day on which treatment was initiated and FH suggests that this outcome is not affected by the timing of treatment initiation, at least under the condition of CH screening and starting treatment described in this paper. While for mental delay it is supposed that some differences do in fact exist in relationship with age at diagnosis [21], our data suggest that the earlier diagnosis does not influence the height outcome.

We confirm that TH and height at puberty onset are the most important factors affecting FH in CH patients. These patients are taller than TH by 2-2.5 cm, in agreement with data previously reported [7]. The height gain found in this study seems in keeping with the described secular trend of 0.6-1 cm/year [35, 36].

The puberty onset occurred within the normal limits in almost all the patients recruited, confirming previous data [3–8]. We did not find any difference between FH and height at puberty onset, suggesting that the pre-pubertal and the pubertal height gain were similar to the normal population.

The prevalence of overweight was higher than the previous French data reported by Léger et al. [20], while the prevalence of obesity was comparable. We did not recruit a control group the evaluate if the prevalence of overweight/obesity was higher than in the normal population, but we think that our findings are in keeping with the Italian data reported by the Italian Health System (http://www.epicentro.iss.it/problemi/obesita/EpidItalia.asp).

The most robust point of this study is the 20-year study period. There are no reports on a so long period allowing us to proper evaluate any change in FH in CH patients born in 80s and 90s. Our data include patients born in the early period of the CH screening program when the diagnosis was done later and the starting treatment based on lower dose of LT4. Nowadays, the treatment starts earlier, with higher doses of LT4, resulting in the early normalization of thyroid hormone levels. The interpretation of our findings should take into consideration the differences in age at diagnosis and in the starting dose and therefore our results may be relevant to infants diagnosed with CH today to a lower extent than expected.

In conclusion, age at puberty onset, linear growth during puberty and final height are normal in congenital hypothyroid patients diagnosed after neonatal screening. The final height is higher than the target height. Despite the large improvement in the screening strategy and the treatment approach final height did not improve in patients born in 80s and 90s, suggesting that the early diagnosis and the treatment strategy do not seem to affect the final height.

## Abbreviations

CH: Congenital hypothyroidism
LT4: Levo-thyroxine
FH: Final height
TH: Target height
SDS: Standard deviation score
SD: Standard deviation
